# New Drugs, Appliances, and Things Medical

**Published:** 1891-06-27

**Authors:** 


### NEW DRUGS, APPLIANCES, AND THINGS MEDICAL.

[All preparations, appliances, novelties, etc., of which a notice is desired, should be sent for The Editor, to care of The Manager, 140, Strand, London, W.C.]

### SANITAS DISINFECTOR.

(SANITAS CO., LIMITED, BETHNAL GREEN, E.)

This is an ingenious arrangement by which the vapour of Sanitas oil may be continuously given off. It consists of an open meshed wirework frame mounted on a flat tin holder, which can be fixed to the wall of a sick-room, W.C., or to the door of an ash bin, &c. Inside this is a mat saturated with Sanitas oil. It provides a constant supply of Sanitas impregnated air, and acts thus as a most efficient deodoriser and disinfectant. The virtues of Sanitas are now well appreciated. It contains large quantities of peroxide of hydrogen, one of the most active disinfecting agents known. Then Sanitas has the advantages of being absolutely non-poisonous, and, at the same time, pleasant to the nose. We believe that in some form or another it is one of the most used of disinfectant agents. The present invention comes at a period when volatile microbe and evil-smelling gas destructors are most serviceable. We have affixed the specimen sent to us to the ash-bin; a fortnight's experience in warm weather has abundantly justified its use.

### MALTO-CARNIS (CAFFYN).

LIQUOR CARNIS CO., 50, HOLBORN VIADUCT.

It will be remembered that quite recently we favourably noticed Caffyn's Liquor Carnis. The company have now put up this preparation in combination with extract of malt and cocoa. It is a very useful form of concentrated food, which is particularly well adapted for those who have undergone or are undergoing excessive tissue waste. It has the advantage of combining the pleasant flavour of cocoa with the nutritious value of the meat juice and the digestive aid of the malt extract. Our investigation shows the presence of large amounts of coagulable albumen, and the usual reactions characteristic of the other ingredients. It is an ingenious and successful attempt to combine agreeable flavour with high food value.

### THE ZONA PATENT ELASTIC OCCASIONAL BELT FOR LADIES.

ZONA COMPANY, 100, CHARING CROSS ROAD, W.C.

A specimen of the above has been submitted to us to report on. It is admirably suited for its purpose, being handy, cleanly (easily washed), and light. It seems to us to be one of those useful articles which ought to have been invented before. Now they are to be had, we can recommend them, and are sure they will only require to be tried to be appreciated. When we add that there are no buttons or pins required, that it weighs half an ounce only, fits closely yet yields to every movement of the body, and is available for sanitary as well as ordinary towels, we think we have said enough.

